# Acute Glossitis

**Published:** 1874-09

**Authors:** James O. Lewis

**Affiliations:** Greenwood, Fla.


### ACUTE GLOSSITIS.

By JAMES O. LEWIS, M.D., of Greenwood, Fla.

*Editor Atlanta Medical and Surgical Journal:*

In the May number of your esteemed Journal I notice the report of a case of glossitis, with its treatment, which reminds me of a case under my observation, in the year 1869, in a negress. The respiration was very labored and difficult, and the impress of despair was depicted in her countenance. She was scarcely able to articulate; unable to lie down; pulse very rapid, and scarcely distinguishable at the wrist; skin very cool, and clammy with moisture. The tongue was enormously swollen, filling the entire mouth and pharynx, and protruding between the teeth. The first impulse was to incise it freely with a knife; but the then existing sentiment of the negro race was different from what it now is, and being quite satisfied that *any* treatment would come to nothing in the existing state of the case, I gave her twenty grains of calomel, and with a small mop cauterized her tongue and fauces as thoroughly as I could with a strong solution of the nitrate of silver, and directed its repetition every six hours. I left a saturated solution of the chlorate of potassium, to be held in the mouth, and swallowed as far as practicable.

I did not visit her again, as I felt quite sure she would be dead before morning. This was late in the afternoon. Two or three days subsequently I learned from her employer that she had improved from the hour I left her, and she made a prompt and perfect recovery. Now, query: What cured her?—calomel, nitrate of silver, the chlorate of potassium, or *nature*, or all combined?

